# Prognostic Role of Subcutaneous and Visceral Adiposity in Hospitalized Octogenarians with COVID-19

**DOI:** 10.3390/jcm10235500

**Published:** 2021-11-24

**Authors:** Max Scheffler, Laurence Genton, Christophe E. Graf, Jorge Remuinan, Gabriel Gold, Dina Zekry, Christine Serratrice, François R. Herrmann, Aline Mendes

**Affiliations:** 1 Division of Radiology, Diagnostic Department, University Hospitals of Geneva, 1205 Geneva, Switzerland; max.scheffler@hcuge.ch (M.S.); jorge.remuinan@hcuge.ch (J.R.); 2 Unit of Clinical Nutrition, Faculty of Medicine, University Hospitals of Geneva, 1205 Geneva, Switzerland; laurence.genton@hcuge.ch; 3 Division of Internal Medicine and Rehabilitation, Department of Rehabilitation and Geriatrics, Faculty of Medicine, University Hospitals of Geneva, 1205 Geneva, Switzerland; christophe.graf@hcuge.ch; 4 Division of Geriatrics, Department of Rehabilitation and Geriatrics, Faculty of Medicine, University Hospitals of Geneva, 1205 Geneva, Switzerland; gabriel.gold@unige.ch (G.G.); francois.herrmann@hcuge.ch (F.R.H.); 5 Division of Internal Medicine for the Aged, Department of Rehabilitation and Geriatrics, Faculty of Medicine, University Hospitals of Geneva, 1205 Geneva, Switzerland; dina.zekry@hcuge.ch (D.Z.); christine.serratrice@hcuge.ch (C.S.)

**Keywords:** subcutaneous fat, visceral fat, chest CT, COVID-19, mortality

## Abstract

Background: We investigated the prognostic significance of visceral and subcutaneous adiposity in octogenarians with COVID-19. Methods: This paper presents a monocentric retrospective study that was conducted in acute geriatric wards with 64 hospitalized patients aged 80+ who had a diagnosis of COVID-19 and who underwent a chest CT scan. A quantification of the subcutaneous, visceral, and total fat areas was performed after segmentations on the first abdominal slice caudal to the deepest pleural recess on a soft-tissue window setting. Logistic regression models were applied to investigate the association with in-hospital mortality and the extent of COVID-19 pneumonia. Results: The patients had a mean age of 86.4 ± 6.0 years, and 46.9% were male, with a mean BMI of 24.1 ± 4.4Kg/m^2^ and mortality rate of 32.8%. A higher subcutaneous fat area had a protective effect against mortality (OR 0.416; 0.183–0.944 95% CI; *p* = 0.036), which remained significant after adjustments for age, sex, and BMI (OR 0.231; 0.071–0.751 95% CI; *p* = 0.015). Inversely, higher abdominal circumference, total fat area, subcutaneous fat area, and visceral fat were associated with worse COVID-19 pneumonia, with the latter presenting the strongest association after adjustments for age, sex, and BMI (OR 2.862; 1.523–5.379 95% CI; *p* = 0.001). Conclusion: Subcutaneous and visceral fat areas measured on chest CT scans were associated with prognosis in octogenarians with COVID-19.

## 1. Introduction

Before the launch of the current vaccination campaigns around the world, older people were the most severely affected by the COVID-19 pandemic [1]. Older patients show reduced muscle strength and an increased proportion of fat tissue [2,3] that is independent of body mass index (BMI) [4]. These body composition characteristics have been described as independent determinants of bad prognosis in the course of COVID-19 by different studies [5,6,7].

Obesity is associated with a higher prevalence of other risk factors that are related to the COVID-19 severity, such as hypertension and diabetes, but is also recognized as a source of chronic inflammation and as a modulator of the immune response [8,9]. Consequently, obesity and the balance between subcutaneous and visceral adiposity may not only increase the susceptibility of acquiring the infection but also the disease severity, including the risk of a cytokine storm. After becoming directly infected by SARS-CoV-2 via ACE receptors [10,11], the adipocytes of the visceral fat in obese patients secrete IL-6 and increase the production and release of leptin, which enhances the proinflammatory state [12,13,14]. Conversely, adiponectin, a protein hormone that is mostly produced by subcutaneous fat but that is abnormally reduced in obese patients counteracts this inflammatory state by reducing the secretion of IL-6 and TNF-α and by increasing the production of anti-inflammatory cytokines by the adipocytes [15,16]. Therefore, central obesity, i.e., high visceral fat, would be one of the main triggers for the underlying exacerbated inflammatory state that is associated with severe COVID-19 [17,18].

Moreover, a high proportion of visceral fat is also observed in patients with normal BMI, raising the question of whether a “global” measure of body composition such as BMI alone or the quantification of subcutaneous and visceral fat would have the strongest relationship with prognosis in older patients.

Thus, we conducted a study to investigate the association of visceral and subcutaneous adiposity as measured by a chest CT scan with the radiological extent of COVID-19 pneumonia and in-hospital mortality in a population of hospitalized older patients. We make the hypothesis that the visceral and subcutaneous adiposity as measured by chest CT scan is associated with poor outcomes in COVID-19.

## 2. Materials and Methods

### 2.1. Design, Setting and Participants

This was a monocentric retrospective study that included patients who were hospitalized in acute wards in the Geriatric Hospital. The Geriatric Hospital was in charge of all of the hospital admissions of older patients with COVID-19 in a geographic region covering an area of approximately five hundred thousand inhabitants. Hospitalized patients with COVID-19 had one or more of these clinical features: (a) pneumonia with a severity assessed by a CURB-65 score ≥ 2; (b) new dependence on oxygen or increase of oxygen needs; (c) a respiratory rate ≥ 20 breaths/minute; (d) decompensated chronic diseases; (e) severely altered general state of health; and (f) deteriorating clinical course.

We admitted 235 patients with SARS-CoV-2 infection from 13 March 2020 to 15 May 2020, all of whom were screened for the presence of COVID-19-related pneumonia by means of chest CT scan. Among them, 64 patients had a chest CT scan performed according to routine clinical practice and recommendations and were included in the present analysis. This subgroup was representative of all of the patients who had been admitted to the hospital according to a feasibility analysis performed before the launch of this study by comparing the patients who underwent a chest CT scan and those who did not.

Of the population presented here, no patient was admitted to the intensive care wards after a shared decision process with the patients and their family members and/or representatives. The only exclusion criterion was refusal to participate in a research study, which was not documented in any medical records; hence, all of the patients who were hospitalized during the study period were included in the analysis.

As outcomes, we defined the extent of COVID-19-associated pneumonia visually as quantified by the chest CT scans and by in-hospital mortality. This study was approved by the local committee for ethics in research (Project-Id: 2020-00819; NCT04385212).

### 2.2. Data Collection

Data regarding the demographics, clinical, and laboratory values were collected based on the information available from the patients’ medical records. Among preexisting comorbidities, we listed the presence of hypertension, diabetes, dyslipidemia, heart failure, chronic obstructive pulmonary disease (COPD), kidney disease, liver disease, active neoplasia, cognitive impairment, Parkinson’s disease, history of stroke, smoking status, and immunosuppression. Several scales and scores based on the clinical data retrieved at hospital admission and clinical data detailed thereafter were also included in the dataset. The cumulative illness rating scale for geriatrics (CIRS-G) measured the chronic medical illness (“morbidity”) burden in 14 individual body systems and assigned grades for each body system that ranged from 0 (no disease) to 4 (very severe) [19]. The total score for this assessment ranges from 0 to 56 points. The confusion assessment method (CAM) was the standard screening tool to detect delirium [20]. The clinical frailty scale (CFS) is a 9-point scale based on clinical judgment that varies from 1 “Very fit” to 9 “Terminally ill” [21]. It has been validated to predict death or the need for institutional care. The functional independence measure (FIM) takes into account physical, psychological, and social functions and was performed within the first 24 h of hospital admission. The scoring system ranges from 18 points (extreme disability) to 126 points (complete independence) [22]. The CURB-65 score is a four-item score that is used to estimate mortality related to community-acquired pneumonia and can help to determine inpatient vs. outpatient treatment [23]. The nutritional risk screening (NRS-2002) score assesses the severity of malnutrition (0–3 points) and the severity of the acute disease (0–3 points), with total scores ranging from 0 to 7, with 3–7 points indicating nutritional risk. An additional point is added for patients who are aged 70 or older [24].

### 2.3. CT Scans Acquisitions, Interpretation and Quantification

All of the chest CT scans were performed using a Somatom AS+ machine (Siemens Healthineers, Erlangen, Germany). An unenhanced chest CT scan was obtained to quantify COVID-19-associated pneumonia and to estimate the degree of consolidation or pleural effusion, and contrast-enhanced studies were performed when there were clinical or biological signs of associated pulmonary embolism. Acquisition parameters were kilovoltage setting, 100–120 kV; pitch, 0.9–1.2; slice thickness, 2 mm with 1 mm increment; automatic tube current modulation (unenhanced scans); and kilovoltage setting, 100–120 kV; pitch, 1.2; slice thickness, 1.5 mm with 1 mm increment; and automatic tube current modulation (CT angiography for research of pulmonary embolism). Patients were in the supine position for all scans, and acquisition took place during end-inspiration breathhold whenever possible. If applicable, the injected contrast medium was Accupaque 350 (GE Healthcare, Oslo, Norway, or GE Healthcare, Cork, Ireland).

**Figure 1:**
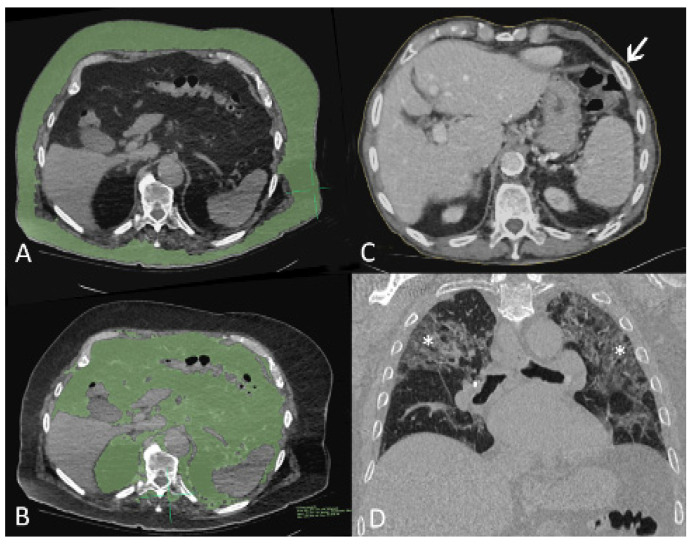
Computed tomography (CT) images of three patients with COVID-19 pneumonia. (**A**,**B**), axial images of 89-year-old man, soft tissue window setting. The first slice caudal to the pleural recesses shows overlay segmented parietal (**A**) and visceral (**B**) fat in green. (**C**) axial image of 93-year-old man, soft tissue window setting. Fine yellow line (arrow) delineates body circumference on the first slice caudal to pleural recesses. (**D**) Coronal-oblique reconstructed image of 86-year-old man in lung window setting shows stage 3 lung infiltrates with ground glass opacities, intralobular septal thickening, and parenchymal bands (asterisks).

For this study, visceral and parietal fat surface measurements were performed by a trained radiologic technologist (J.R.) using the Osirix MD application for Mac (Version 12.0.1, Pixmeo, Bernex, Switzerland) and using the 2D segmentation region of interest (ROI) growth tool while adjusting the density intervals around the chosen starting points. The segmentations were performed on the first abdominal slice caudal to the deepest pleural recess on a soft-tissue window setting. Illustrative examples are shown in Figure 1A,B. Upper abdominal circumference was determined by the same person on the same slice using the Osirix MD closed polygon ROI tool, as shown in Figure 1C. The degree COVID-19-induced lung affection was assessed for all patients by a staff radiologist (M.S.) on a PACS workstation in a lung window setting and using multiplanar reconstruction. Stage 1 was assigned if 0–25% of the lung parenchyma was affected, stage 2 was assigned for 26–50% affection, stage 3 was assigned for 51–75% affection, and stage 4 was assigned for >75% affection. An example for stage 3 affection is provided in Figure 1D.

### 2.4. Statistical Analysis

Categorical variables were described as absolute numbers and proportions, while continuous variables were described as means and standard deviations. We performed a two-group comparison (survivors vs. non-survivors) using the Chi-square test or the *t*-test, depending on the variable type. The Mann–Whitney u test was used to compare the ordinal variables. Results were considered statistically significant when *p* values were <0.05.

Regarding outcomes, we used ordered logistic regression models and logistic regression models to investigate the relationship between the fat measures and the radiological extent of COVID-19 pneumonia as mentioned above and in-hospital mortality, respectively. Univariate (Model 1) and multivariate analysis were performed, with adjustments being made for age and sex (Model 2) and age, sex, and BMI (Model 3) for each measure of adiposity. Results were expressed as the Odds Ratio (OR) followed by the 95% confidence interval and its respective *p*-value and pseudo-R^2^, which is the proportion of variance that is explained by the model. Then, we studied the association of our best-adjusted model with in-hospital mortality by calculating the area under the receiver operating characteristic (AUC) curve. Statistical analysis was performed using the Stata^®^ software (version 16.1, StataCorp 2019, College Station, TX, USA).

## 3. Results

### 3.1. Characteristics of the Study Population

In the group of 64 patients who underwent chest CT scans, the mean age was of 86.4 ± 6.0 years, and 46.9% were male. The time from the beginning of symptom onset to hospital admission was 3.5 ± 3.1 days, and the mean LOS in acute care was 12.5 ± 5.6 days. The patients were frequently frail (CFS: 5.7 ± 1.8), with a high disease burden and functional impairment according to their CIRS-G (19.3 ± 6.1) and FIM scores (75.5 ± 31.1). The most prevalent comorbidities were hypertension (68.8%) followed by cognitive disorders (51.6%), dyslipidemia (40.6%), and heart failure (40%). It is worth noting that the majority of patients were categorized as being of a normal weight (46%), followed by the categories of overweight (28.6%) and underweight (15.9%). A minority of patients in this cohort was classified as obese (9.5%) according to BMI. Moreover, 78.2% of the patients had a nutritional risk according to the NRS (≥3) performed at admission. Pulmonary embolism was detected in one patient in the non-survivor group (*p* = 0.328).

The deceased patients (*n* = 21) were mainly male (76.2% vs. 32.6%; *p* = 0.001) and had a shorter LOS than the survivors (10.0 ± 6.1 vs. 13.7 ± 5.0; *p* = 0.025). A detailed description of the characteristics of the population is shown in Table 1.

### 3.2. Adiposity Measures and the Radiological Extent of COVID-19 Pneumonia

More than half of the patients in this cohort presented stage 1 (0–25%) lung involvement at chest CT, with no differences being determined between the survivors and non-survivors. In the univariate analysis, age, sex, and BMI were not significantly associated with the extent of pneumonia in the chest CT scans. On the other hand, we observed a significant association among all four measures of adiposity (upper abdominal circumference, total fat area, subcutaneous fat area, and visceral fat area) with the extent of COVID-19 pneumonia in the univariate and multiple models. Specifically, each increase of 1 dm^2^ in the visceral fat area increased the risk of being in a category of more severe pulmonary involvement by more than 2.6 times after adjustments for age, sex, and BMI. Visceral fat presented the strongest association with this outcome, as presented in Table 2.

### 3.3. Adiposity Measures and In-Hospital Mortality

Of the total 64 patients, 21 (32.8%) died during hospitalization. Survivors had a higher subcutaneous fat area than the non-survivors did (142.7 ± 85.0 vs. 92.6 ± 81.1; *p* = 0.028) no significant difference was detected for the visceral and total fat areas, nor were any significant differences found for the upper abdominal circumference (Table 1).

**Figure 2:**
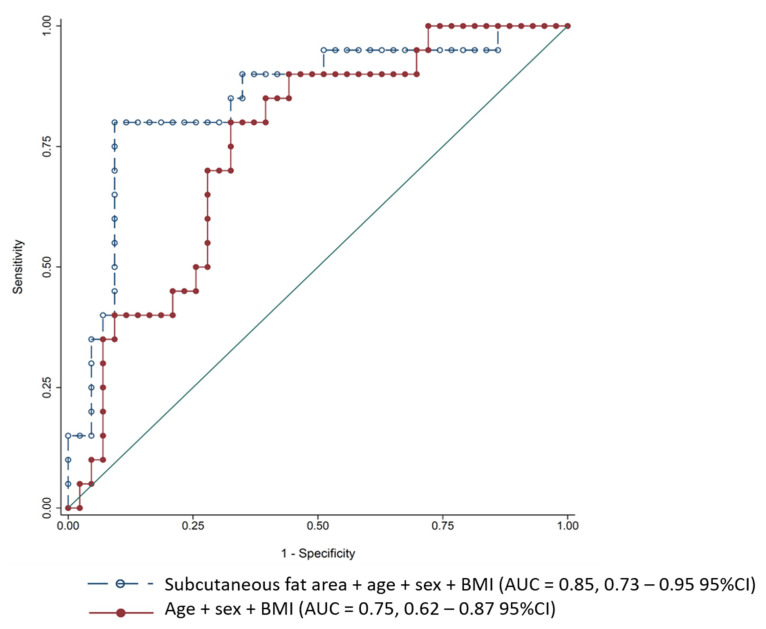
Receiver operating characteristic (ROC) curves for mortality prediction. Abbreviations: BMI = body mass index; AUC = area under the receiver operating characteristic curve; CI = confidence interval.

A higher subcutaneous fat area had a protective effect against mortality, as each increase by 1 dm^2^ reduced the risk of dying by approximately 59% in the univariate analysis model. This association remained significant in the multivariate models, with an even higher strength association being observed after adjustments for age, sex, and BMI (OR 0.231; 0.071–0.751 95% CI; *p* = 0.015). A ROC curve was computed for this model (subcutaneous fat adjusted for age, sex, and BMI) and was compared to age, sex, and BMI alone using the likelihood ratio test. We detected an 11% gain in explaining the variance of the outcome (mortality) to the effect of the subcutaneous fat area only (Figure 2).

Although weaker than the effect of the subcutaneous fat area, higher upper abdominal circumference and the total fat area also presented an association with survival in the multivariate models. There was no association with the visceral fat area or BMI with in-hospital mortality (Table 3).

## 4. Discussion

This study, which was conducted in a population of hospitalized octogenarians with COVID-19, demonstrated that the subcutaneous and visceral fat areas had a significant effect on prognosis, albeit different effects. While a higher subcutaneous fat area was protective against mortality, with visceral fat showing no significant affect against morality, a higher proportion of visceral fat was strongly associated with greater radiological COVID-19-related pneumonia severity. Additionally, we demonstrated the feasibility and clinical relevance of body composition measures assessed by chest CT scans in this specific population.

COVID-19 leads to significant changes in body composition. A post hoc analysis performed in a population consisting mainly of patients in the overweight/obesity categories (70%) showed that among survivors, nearly 30% lost 5% or more of their body weight, with those presenting acute respiratory distress syndrome (ARDS) losing up to 18% of their body weight [25]. The systemic inflammatory response that occurs with severe infection triggers catabolic states, followed by increased lipolysis and skeletal muscle wasting [26]. Drawing a parallel with the results of our study, we hypothesize that in these largely non-obese frail patients, subcutaneous fat is a strategic source of energy supply, thus explaining the protective effect observed against in-hospital mortality. Furthermore, subcutaneous adipocytes have anti-inflammatory properties that are mediated by the secretion of adiponectin. This hormonal balance between subcutaneous and proinflammatory visceral adipocytes may be another important mechanism explaining our results [27].

We did not find any relationship between the visceral fat area and mortality in this population. We believe the special characteristics of our very old study population may explain the differences between our results and previous reports with a similar methodology [6,7,18,28,29,30]. We built up new information on the role of adiposity in very old patients that does not preclude the notion that a high proportion of visceral fat, especially in younger obese patients, triggers inflammation and more severe disease, as established by the previous evidence.

In our study, a higher visceral fat area was strongly associated with g SARS-CoV-2 having greater lung involvement at hospital admission. It is worth noting that our CT scans were performed relatively early, close to hospital admission, and they do not reflect the overall severity of the disease during follow-up, which is corroborated by the fact that more than half of the patients presented with stage 1 pneumonia. Interestingly, our results raise the question of whether a higher visceral fat area could play a role as a marker of early pulmonary involvement in COVID-19.

One of the main strengths of this study was its participants, with this study being the first in this domain to specifically investigate a group of 80+ patients to date. The use of chest CT scans allowed us to adapt a routinely performed diagnostic test to add new measures of body composition, specifically visceral and subcutaneous fat mass.

However, this study has several limitations. Only one measure of body composition was performed at the beginning of hospitalization in the subgroup of patients, which does not allow us to conclude the impact of the changes that took place during the hospital stay. Furthermore, the obese category was only represented by a few patients in this study, meaning that this should be the object of further study in this research field. Additionally, the small number of participants and deaths may have contributed to the lack of power in some models as well as to the lack of power in the comparison between groups, as the survivors and non-survivors had similar morbidity profiles. Finally, the radiological features of COVID-19-related pneumonia do not necessarily correlate with the severity of respiratory status, which should be integrated into multivariate models in future analysis.

## 5. Conclusions

In conclusion, the subcutaneous and visceral fat areas measured on routinely performed chest CT scans presented a significant prognostic role in a population of octogenarians with COVID-19. Importantly, these specific body composition measures were more relevant prognostic markers than BMI.

## Figures and Tables

**Table 1:** Characteristics of the study population.

Characteristics	Intra-Hospital Death			
	Total	No	Yes	*p* Value
*N*	64	43	21	
Age, year	86.4 ± 6.0	86.3 ± 5.8	86.7 ± 6.6	0.797
Male sex	30 (46.9%)	14 (32.6%)	16 (76.2%)	0.001
Time from symptoms to hospital admission, day	3.6 ± 3.1	4.1 ± 3.5	2.6 ± 1.7	0.026
Length of stay, day	12.5 ± 5.6	13.7 ± 5.0	10.0 ± 6.1	0.025
FIM	75.5 ± 31.1	82.5 ± 28.4	52.9 ± 29.8	0.005
CFS	5.7 ± 1.8	5.2 ± 1.7	6.8 ± 1.4	<0.001
CIRS-G	19.3 ± 6.1	18.3 ± 6.4	21.1 ± 5.0	0.060
CAM	13 (20.6%)	6 (14.0%)	7 (35.0%)	0.092
CURB-65				0.648
1	10 (15.6%)	8 (18.6%)	2 (9.5%)	
2	29 (45.3%)	20 (46.5%)	9 (42.9%)	
3	23 (35.9%)	14 (32.6%)	9 (42.9%)	
4	2 (3.1%)	1 (2.3%)	1 (4.8%)	
ARDS	16 (25.0%)	3 (7.0%)	13 (61.9%)	<0.001
BMI kg/m^2^	24.1 ± 4.4	23.6 ± 4.3	25.1 ± 4.6	0.217
BMI kg/m^2^				0.247
<20	10 (15.9%)	8 (18.6%)	2 (10.0%)	
20–24.9	29 (46.0%)	21 (48.8%)	8 (40.0%)	
25–29.9	18 (28.6%)	10 (23.3%)	8 (40.0%)	
30+	6 (9.5%)	4 (9.3%)	2 (10.0%)	
NRS				0.315
0–2	14 (21.9%)	13 (30.2%)	1 (4.8%)	
3–4	20 (31.3%)	10 (23.3%)	10 (47.6%)	
5–7	30 (46.9%)	20 (46.5%)	10 (47.6%)	
Hypertension	44 (68.8%)	31 (72.1%)	13 (61.9%)	0.566
Dyslipidemia	26 (40.6%)	16 (37.2%)	10 (47.6%)	0.588
Heart Failure	24 (40.0%)	13 (32.5%)	11 (55.0%)	0.105
Diabetes	14 (21.9%)	9 (20.9%)	5 (23.8%)	>0.99
Kidney disease	14 (21.9%)	10 (23.3%)	4 (19.0%)	>0.99
Liver disease	4 (6.3%)	2 (4.7%)	2 (9.5%)	0.592
COPD	4 (6.3%)	3 (7.0%)	1 (4.8%)	>0.99
Smoking				0.789
No smoking	46 (71.9%)	32 (74.4%)	14 (66.7%)	
Past	15 (23.4%)	9 (20.9%)	6 (28.6%)	
Present	3 (4.7%)	2 (4.7%)	1 (4.8%)	
Stroke	16 (25.8%)	11 (26.2%)	5 (25.0%)	>0.99
Parkinson disease	3 (4.8%)	2 (4.7%)	1 (5.0%)	>0.99
Cognitive disorders	33 (51.6%)	23 (53.5%)	10 (47.6%)	0.791
Known swallowing disorders	4 (6.3%)	2 (4.7%)	2 (9.5%)	0.592
Active neoplasia	5 (7.8%)	3 (7.0%)	2 (9.5%)	>0.99
Immunosuppression	3 (4.7%)	2 (4.7%)	1 (4.8%)	>0.99
Albumin	35.5 ± 8.0	35.7 ± 8.8	34.8 ± 5.2	0.641
C-Reactive Protein	56.2 ± 66.1	48.0 ± 41.2	73.9 ± 100.2	0.279
Lymphocytes nb-abs	1.3 ± 1.2	1.3 ± 1.3	1.1 ± 0.7	0.395
Radiological Measures				
Extent of COVID-19 pneumonia				0.813
0–25%	33 (54.1%)	22 (53.7%)	11 (55.0%)	
26–50%	13 (21.3%)	10 (24.4%)	3 (15.0%)	
51–75%	11 (18.0%)	7 (17.1%)	4 (20.0%)	
76–100%	4 (6.6%)	2 (4.9%)	2 (10.0%)	
AC (mm)	714.0 ± 196.3	743.9 ± 183.6	652.9 ± 211.6	0.101
TF (mm^2^)	267.5 ± 143.0	285.4 ± 142.6	231.1 ± 140.1	0.156
SF (mm^2^)	126.2 ± 86.4	142.7 ± 85.0	92.6 ± 81.1	0.028
VF (mm^2^)	141.3 ± 84.0	142.7 ± 81.9	138.5 ± 90.2	0.858

Abbreviations: FIM = functional independence measure; CFS = clinical frailty scale; CIRS-G = cumulative illness rating scale for geriatrics; CAM = confusion assessment method; ARDS = acute respiratory distress syndrome; BMI = body mass index; NRS = nutritional risk screening; TF = total fat area (mm^2^); AC = upper abdominal circumference (mm); SF = subcutaneous fat area (mm^2^); VT = visceral fat area (mm^2^).

**Table 2:** Univariate and multiple ordered logistic regression models for the association with the extent of COVID-19 pneumonia.

Extent of COVID-19 Pneumonia	Model 1—Univariate				Model 2—Adjusted For Age and Sex				Model 3—Adjusted for Age, Sex and BMI			
	OR	95% CI	*p* Value	R^2^	OR	95% CI	*p* Value	R^2^	OR	95% CI	*p* Value	R^2^
Age	1.011	0.932–1.095	0.794	0.5%								
Male sex	1.678	0.637–4.416	0.294	0.8%								
BMI	1.004	0.905–1.114	0.934	0.1%	0.001	0.001–0.055	0.985	0.5%				
Upper abdominal circumference	1.041	1.014–1.068	0.003	7.3%	1.041	1.014–1.069	0.002	8.0%	1.042	1.015–1.071	0.002	8.2%
Total fat area	1.766	1.230–2.537	0.002	7.9%	1.806	1.249–2.609	0.002	9.1%	1.851	1.27–2.695	0.001	9.5%
Subcutaneous fat area	1.817	1.078–3.060	0.025	3.7%	1.856	1.094–3.149	0.022	4.7%	1.917	1.124–3.271	0.017	4.8%
Visceral fat area	2.692	1.461–4.961	0.001	7.9%	2.784	1.489–5.206	0.001	9%	2.862	1.523–5.379	0.001	9.3%

Abbreviations: BMI = body mass index (kg/m^2^); TF = total fat area (dm^2^); AC = upper abdominal circumference (dm); SF = subcutaneous fat area (dm^2^); VT = visceral fat area (dm^2^).

**Table 3:** Univariate and multiple logistic regression models for the association of in-hospital mortality.

	Model 1—Univariate				Model 2—Adjusted for Age and Sex				Model 3—Adjusted for Age, Sex and BMI			
In-Hospital Mortality	OR	95% CI	*p* Value	R^2^	OR	95% CI	*p* Value	R^2^	OR	95% CI	*p* Value	R^2^
Age	1.012	0.927–1.105	0.784	0.9%								
Male sex	6.628	2.017–21.781	0.002	13.8%								
BMI	1.083	0.958–1.223	0.201	2.1%	1.102	0.956–1.271	0.182	15.3%				
Upper abdominal circumference	0.975	0.946–1.003	0.086	3.9%	0.953	0.914–0.994	0.025	23.1%	0.95	0.913–0.989	0.013	25.2%
Total fat area	0.746	0.496–1.119	0.158	2.7%	0.579	0.342–0.982	0.043	19.8%	0.578	0.336–0.993	0.047	21.2%
Subcutaneous fat area	0.416	0.183–0.944	0.036	6.8%	0.219	0.067–0.717	0.012	25.5%	0.231	0.071–0.751	0.015	26.4%
Visceral fat area	0.941	0.500–1.769	0.85	0.4%	0.783	0.385–1.591	0.499	14.4%	0.78	0.376–1.617	0.505	15.8%

Abbreviations: BMI = body mass index (kg/m^2^); TF = total fat area (dm^2^); AC = upper abdominal circumference (dm); SF = subcutaneous fat area (dm^2^); VT = visceral fat area (dm^2^).

## Data Availability

The data that support the findings of this study are available from the corresponding author (A.M.) upon reasonable request.
